# Postural Control Differences between Patients with Posterior Tibial Tendon Dysfunction and Healthy People during Gait

**DOI:** 10.3390/ijerph19031301

**Published:** 2022-01-24

**Authors:** Junsig Wang, L. Daniel Latt, Robert D. Martin, Erin M. Mannen

**Affiliations:** 1 Department of Sports Medicine, Kyung Hee University, Yongin-si 17014, Gyeonggi-do, Korea; 2 Department of Orthopaedic Surgery, University of Arkansas for Medical Science, Little Rock, AR 72205, USA; RDMartin@uams.edu (R.D.M.); erinmannen@boisestate.edu (E.M.M.); 3 Department of Orthopaedic Surgery, University of Arizona, Tucson, AZ 85724, USA; dlatt@ortho.arizona.edu; 4 Mechanical and Biomedical Engineering Department, Boise State University, Boise, ID 83725, USA

**Keywords:** postural control, PTTD, center of pressure, time-to-boundary, gait

## Abstract

Background: Patients with posterior tibial tendon dysfunction (PTTD) may exhibit postural instability during walking likely due to a loss of medial longitudinal arch, abnormal foot alignment, and pain. While many studies have investigated gait alterations in PTTD, there is no understanding of dynamic postural control mechanisms in this population during gait, which will help guide rehabilitation and gait training programs for patients with PTTD. The purpose of the study was to assess dynamic postural control mechanisms in patients with stage II PTTD as compared to age and gender matched healthy controls. Methods: Eleven patients with stage II PTTD (4 males and 7 females; age 59 ± 1 years; height 1.66 ± 0.12 m; mass 84.2 ± 16.0 kg) and ten gender and age matched controls were recruited in this study. Participants were asked to walk along a 10 m walkway. Ten Vicon cameras and four AMTI force platforms were used to collect kinematic and center of pressure (COP) data while participants performed gait. To test differences between PTTD vs. control groups, independent *t*-tests (set at α < 0.05) were performed. Results: Patients with PTTD had significantly higher double stance ratio (+23%) and anterior-posterior (AP) time to contact (TTC) percentage (+16%) as compared to healthy control. However, PTTD had lower AP COP excursion (−19%), AP COP velocity (−30%), and medial-lateral (ML) COP velocity (−40%) as compared to healthy controls. Mean ML COP trace values for PTTD were significantly decreased (−23%) as compared to controls, indicating COP trace for PTTD tends to be closer to the medial boundary than controls during single-support phase of walking. Conclusion: PTTD patients showed more conservative and cautious postural strategies which may help maintain balance and reduce the need for postural adjustment during PTTD gait. They also showed more medially shifted COP patterns than healthy controls during single-support phase of walking. Dynamic postural control outcomes could be used to develop effective gait training programs aimed at alleviating a medial shift of COP (everted foot) for individuals with PTTD in order to improve their functionality and gait efficiency.

## 1. Introduction

Posterior tibial tendon dysfunction (PTTD) is a common and debilitating tendinopathy of the posterior tibial tendon and affects 3.3% of women over the age of 40 [1]. During gait, the posterior tibial tendon plays a crucial role in stabilizing and elevating the medial arch, plantarflexing the ankle, and inverting the foot [2]. Patients with PTTD likely suffer from gait deficit and decreased ambulatory function due to a loss of the medial longitudinal arch, abnormal foot alignment, and pain [3,4,5]. Numerous studies have found differences in multi-segmental foot kinematics between patients with PTTD and healthy controls during gait alterations [6,7,8,9]. For example, these studies found that PTTD patients had increased forefoot dorsiflexion, forefoot abduction, hindfoot plantarflexion, and hindfoot eversion. However, differences in postural control mechanisms during gait have not been characterized. An understanding of the dynamic postural control in patients with PTTD could help identify fall-related walking deficits and help evaluate the effectiveness of surgical and non-surgical interventions aimed at improving gait function.

Center of pressure (COP) movements that reflect changes in the center of mass (COM) have been commonly used in the assessment of postural control. Greater COP movement during quiet standing is likely associated with poor balance control and an increased risk of falls. Previously, only one study has investigated postural control in patients with PTTD during single leg standing, suggesting that PTTD patients had a lower success rate of single-leg balance tasks and increased COP excursions compared to healthy controls [10]. However, it is not known whether the patients with PTTD have altered postural control mechanisms during dynamic tasks such as walking. Therefore, there is a great need to investigate postural control in PTTD patients which can provide valuable insight into gait efficiency and fall risks.

Time-to-contact (TTC), also known as time-to-boundary, has also been used to evaluate postural stability [11,12]. TTC is the estimated time it takes the COP to reach the boundary of foot [12]. TTC includes both spatial and temporal (velocity and acceleration) aspects of postural control relative to the base of support [11]. A modified TTC method has been proposed to assess postural stability during gait since the standard TTC analysis is challenging to apply to dynamic activities. Specifically, the COP must leave the boundary of one foot and shift to the other foot as human body progresses and thus a modification to the standard TTC is required to identify postural demands when individuals have to make postural adjustments in order to maintain balance during walking. While most previous studies on the standard TTC have investigated quiet standing, the modified TTC method allows the evaluation of postural stability during gait. This modified TTC was effective in evaluating postural stability in individuals with anterior cruciate ligament reconstruction during stair negotiation and healthy adults when carrying an asymmetric load during gait [13,14]. Thus, this novel TTC approach may be of value to investigations of postural control mechanisms for PTTD patients during walking.

Structured classification systems are used when considering the diagnosis and treatment of PTTD. Stage II involves increased tendon length with progressed hindfoot valgus, forefoot abduction, and loss of the medial longitudinal arch [15,16]. Thus, it is of value to evaluate gait function of stage II PTTD when considering etiology and functionality of PTTD. While previous studies demonstrated frontal plane gait alterations such as increased forefoot abduction and hindfoot eversion for stage II PTTD patients compared to healthy controls, no study has yet investigated dynamic postural control for PTTD during gait. These findings (changes in frontal plane foot kinematics) support the idea that patients with PTTD would have postural control challenges, particularly in medial-lateral (ML) direction.

The purpose of the current study is to assess postural control mechanisms during gait in patients with stage II PTTD as compared to age and gender matched healthy controls. We hypothesized that ML TTC percentage, ML COP excursion, and ML COP velocity would be decreased for PTTD patients as compared to healthy controls during the single-support phase of walking.

## 2. Methods

### 2.1. Participants

Participants were recruited from two academic tertiary care ambulatory orthopedic clinics: University of Arkansas for Medical Sciences (UAMS) and University of Arizona (UA) by two foot and ankle specialists (R.D.M. and L.D.L.). Eleven PTTD patients with unilateral stage II PTTD (four patients from UAMS and six patients from UA) agreed to participate in the study (Table 1). Participants were included if they met the following criteria: (1) stage II PTTD, (2) no other lower extremity disorder or surgery, (3) no comorbidities that impact pain and function, (4) a BMI of less than 40 (to exclude “severe obesity”) [17], and (5) able to walk 10 m unassisted. Each participant completed the informed consent process approved by either the UA and UAMS institutional review boards. Eleven age- and gender-matched healthy controls were recruited to compare to the 11 PTTD patients (Table 1). The healthy controls all had a normal foot structure and hindfoot posture during standing [18,19].

### 2.2. Experimental Procedures

**Figure 1:**
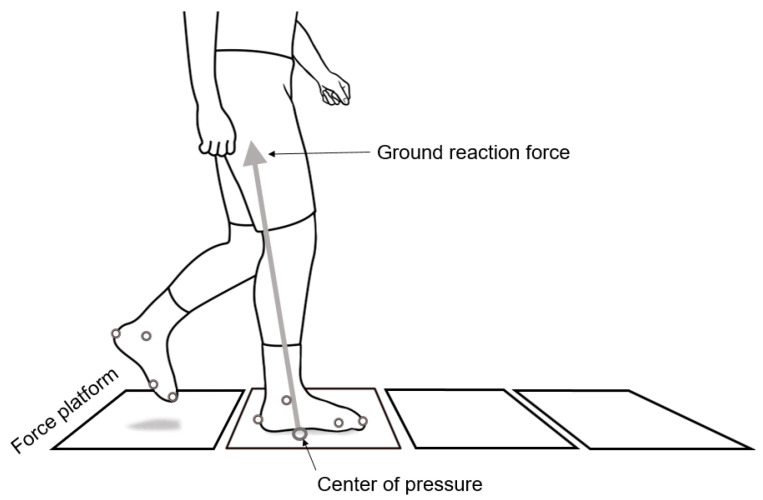
Experimental setup (at UAMS) showing four force platforms which were used to assess center of pressure (COP) movement during single-support phase (only one limb is in contact with the ground) of walking.

Three-dimensional kinematic data were collected using 10 cameras (Vicon, Oxford, UK) operated at 100 Hz. Twelve makers were placed on both feet: bilateral great toe, heel, medial midfoot, lateral midfoot, medial malleolus, and lateral malleolus. Four force platforms (at UAMS; dimension: 40 cm × 60 cm; AMTI, Watertown, MA, USA) or one force platform (at UA; dimension: 60 cm × 90 cm; AMTI, Watertown, MA, USA) were used to collect force and COP data operating at 1000 Hz (Figure 1). Video and force platform data were synchronized using Vicon Nexus (Vicon, Oxford, UK). The participants were instructed to walk on a 10-m walkway at their preferred pace, until three trials (three steps) of complete data were captured where a foot contact (for the affected limb) within the borders of one of the four force platforms (or one lager force platform) was required for a trial to be deemed complete.

### 2.3. Data Processing

**Figure 2:**
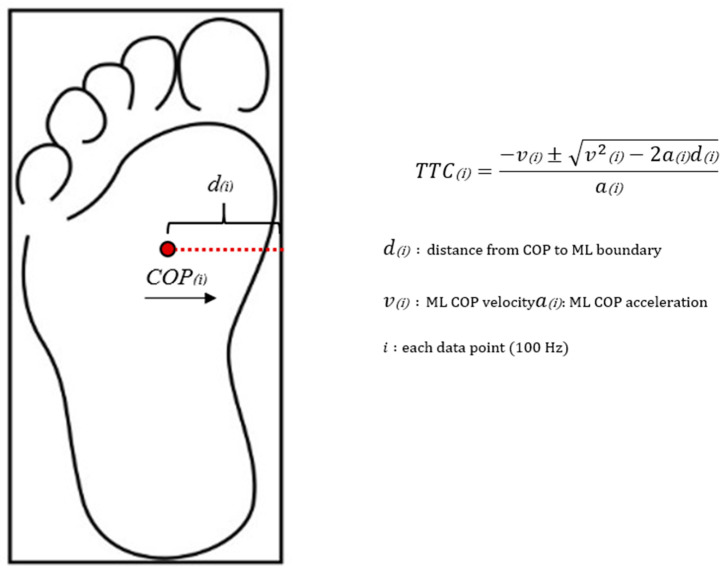
Illustration of the rectangular boundary of the foot and the Time-to-Contact (TTC) calculation. v and a (COP velocity and acceleration) were calculated using the first central difference method.

Single-support and double-support phases were determined using the vertical ground reaction force and toe velocities [20]. Toe velocities were used to define the gait events for the contralateral limb because of the limited number of force plates or off-foot contact within the force platform. Double stance ratio was calculated as a ratio of double stance time to single-support time. COP excursions and mean COP velocities in both anterior-posterior (AP) and ML directions were determined during single-support phases for the affected foot. The COP velocities and accelerations were calculated utilizing the first central difference method. Rectangular boundaries for each foot (Figure 2) were determined using the heel, toe, medial midfoot, and lateral midfoot markers.

**Figure 3:**
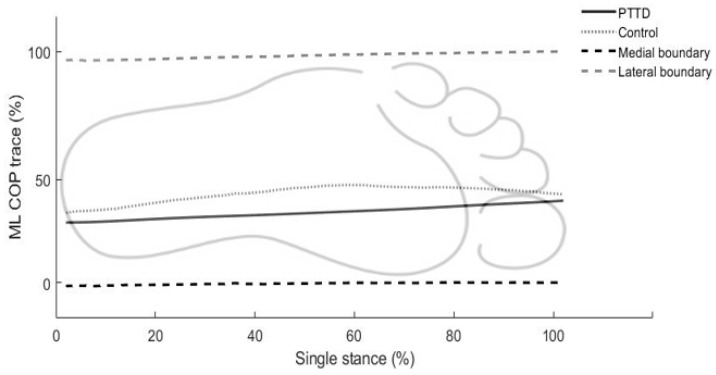
Illustration of normalized COP trace relative to the medial-lateral boundary of the foot: 0% of the COP trace indicates that COP is close to the medial boundary, 100% indicates COP is close to the lateral boundary, and 50% indicates COP is in the middle of the medial-lateral boundary.

ML COP positions, velocities, and accelerations were input into the equation in Figure 2 to calculate ML TTC. Since the COP shifts between the boundaries of each foot during walking, a modified version of TTC was utilized [20,21]. Briefly, TTC was calculated at each data point and then compared to the remaining single-support time. If the TTC was less than the remaining single-support time, then the TTC value was stored for that time point, indicating a postural adjustment was required during single-support phase. For instance, if the TTC is 0.20 s and the remaining single-support time is 0.25 s, the TTC (0.20 s) was stored, which indicates a postural adjustment is needed before double support phase when the participants could utilize a larger base of support for COP control. Conversely, if the TTC was greater than the remaining single-support time, then the TTC was set to the remaining single-support time, indicating double support begins before a postural adjustment was needed. TTC percentage was then calculated by dividing average TTC during single-support phase by one-half of single-support time. Thus, a TTC percentage of 100% indicated that no postural adjustment was required during single-support phase. In addition, mean ML COP trace was determined during single-support phase and then normalized to ML boundaries (Figure 3), which might be of interest due to changes in the PTTD foot structure (e.g., loss of the medial longitudinal arch and everted hind foot). If the normalized ML COP trace is close to 0% or 100%, COP is reaching the medal or lateral boundary. If the ML COP trace is 50%, COP is exactly in the middle of the ML boundary.

### 2.4. Statistical Analyses

There were eight dependent variables: double stance ratio, AP and ML COP excursions, mean AP and ML COP velocities, AP and ML TTC percentages, and mean ML COP trace. Double stance ratio and COP-based parameters were calculated using custom Matlab code (Mathworks Inc., Natik, MA, USA). We compared the patients with PTTD to healthy controls to test our hypothesis. Unpaired *t*-tests were performed where appropriate using the SPSS statistical package (IBM SPSS Statistics for Windows, Version 23.0. IBM Corp.: Armonk, NY, USA). For comparisons of statistical significance, Cohen’s d effect sizes were calculated and interpreted as follows [22]: 0.20–0.49 = small effect; 0.50–0.79 = medium effect and ≥0.8 large effect. The level of statistical significance for all tests was set at *p* < 0.05.

## 3. Results

PTTD patients had significantly higher double stance ratio and AP TTC percentage (+23% and +16%; *p* = 0.009 and *p* = 0.009) compared to healthy controls, indicating increased double stance time and decreased AP postural adjustments (Table 2). In addition, PTTD patients exhibited limited COP movement during single-support phase of walking as evidenced by decreased AP COP excursion (−19%; *p* = 0.020), AP COP velocity (−30%; *p* < 0.001), and ML COP velocity (−40%; *p* = 0.018) compared to the controls. The patients also showed decreased ML COP trace (−23%; *p* = 0.022) compared to the healthy controls, which indicates medially shifted COP movement during single-support phase of walking. There were no other differences in other COP parameters (ML COP excursion and ML TTC percentage) between PTTD and healthy control groups.

## 4. Discussion

The purpose of the study was to assess postural control mechanisms in patients with stage II PTTD as compared to age and gender matched healthy controls. Contrary to our hypothesis, there was no difference in ML COP TTC percentage between PTTD and healthy control groups, which indicates there is no differences in ML postural control between the two groups. Additionally, we found significantly increased AP COP TTC for PTTD patients as compared to healthy control. These results indicate that PTTD patients needed decreased AP postural adjustments during single-support phase of walking. In order to decrease the need for postural adjustments during gait, the patients probably required other compensatory strategies such slower COP movement and decreased single-support time. By utilizing these conservative postural control strategies, the PTTD patients might be able to maintain postural stability and decrease the demand on postural adjustments compared to healthy controls.

The double stance ratio is another important parameter in this study as other COP parameters were evaluated only during single-support phase of walking. The PTTD patients decreased single-support time (and increased double stance time) as compared to the controls. Increased proportion of double stance phase has been found in balance challenged populations such as patients with knee and hip osteoarthritis and elderly adults, which is indicative of a “cautious and conservative” postural control strategy [23,24,25]. In addition, Brodsky et al. reported that patients with reconstruction for PTTD still had decreased single-support as compared to healthy control [5]. Thus, PTTD patients likely utilize this conservative strategy to decrease the need for postural control with decreased single-support phase (when only one limb is available for postural adjustments), which may help maintain postural stability during unstable gait.

Other differences in COP movements were also found between PTTD and healthy control groups including AP COP excursion and velocity which were decreased for PTTD compared to healthy controls. In general, COP characteristics are closely related to foot function [26]. Similarly, slower COP control during stance of walking was found for patients with first metatarsophalangeal joint osteoarthritis as compared to healthy controls [27]. Restricted AP COP movement may be related to diminished weight transfer during single-support phase, which can result from changes in mid/hind foot structure with decreased rigidity and unlocked bones [28]. Additionally, previous studies found that PTTD patients had decreased hindfoot dorsiflexion during stance of walking, which is also associated with the unlocking bones in a flexible foot [6,8]. Therefore, the PTTD patients likely have an inability to progress COP movements in AP direction, which may lead to lower AP COP excursion and velocity in order to maintain postural stability with a flexible foot by limiting weight transfer during the stance phase of gait.

The patients with PTTD were also found to have decreased ML COP movement as compared to the healthy controls. While comparable data are not available for PTTD postural control mechanisms during gait, Kulig et al. reported that patients with stage I or II PTTD (who were able to complete the single limb balance test) showed increased ML COP excursion during single limb stance test (quiet standing) [10]. Conversely, we found that PTTD patients decreased COP movements (AP and ML COP velocities) with reduced single-support phase of walking. Accordingly, one possible reason for the difference in findings could be due to static versus dynamic conditions, which demonstrates patients with PTTD restricted ML COP movements toward the medial boundary of the foot as evidenced by decreased ML trace (Table 1 and Figure 3). Stage II PTTD patients have progressed hindfoot valgus, forefoot abduction, and loss of the medial longitudinal arch [15,16]. As with these changes in foot structure, numerous studies have demonstrated that PTTD patients had increased eversion of hindfoot and increased abduction of forefoot during gait [7,8,9,29]. Increased forefoot abduction and hindfoot eversion could lead to a medial shift of the COP with increased medial foot loading during weight transfer (single-support phase of walking). In addition, our finding of the decreased ML COP trace (toward medial boundary of the foot) supports this idea and the PTTD patients controlled their COP medially (close to medial boundary of the foot), which may lead to limited ML COP movements and further decreased postural stability during walking.

The current study had the following limitations. First, the COP measures that we used did not reflect whole-body postural control. Thus, without additional kinematic data, we do not know where in the body the postural changes were being made. Second, there is a significant difference in gait speed between the two groups, which can be a potential confounder. However, the speed effect on postural stability is more pronounced at 40% faster and slower than preferred gait speed. The speed effect may be negligible since PTTD patients (0.92 m/s) tend to walk only 20% slower than the healthy controls (1.13 m/s). Third, we analyzed three trials of single-support phases of walking (three steps), so the patients may not have achieved a repeatable postural control pattern. Fourth, partly due to the recent limitations in human subject data collection due to the pandemic, the sample size is small (11 PTTD patients vs. 11 healthy controls). Although we found large effect sizes for most of the significant differences, we did not correct for the multiple statistical analyses performed which may limit generalizability. Fifth, there was a considerable difference in BMI between the PTTD and healthy control groups, which may impact COP variables (Table 1). Thus, further BMI matched control data are needed to rule out potential differences between the groups. Lastly, we did not include muscle strength and proprioception measures and thus we cannot comment on muscle strength and sensory function for PTTD postural control.

## 5. Conclusions

We found a variety of significant changes in dynamic postural control between patients with stage II PTTD and healthy controls. The patients with PTTD utilized increased double support time and slower COP movements in order to maintain postural stability and reduce the demand on postural adjustment during the single-support phase of gait. Their COP movements were shifted to the medial boundary, which could be due to changes in their foot structure. These findings should be considered when developing and improving surgical and non-surgical interventions. For instance, conservative care (e.g., bracing and therapy) should aim to alleviate the medial shift of COP to improve gait function and postural control for PTTD patients. In addition, our findings may help understand surgical efficiency by determining if surgical correction of foot resolves medially shifted and restricted COP movement. Thus, further studies should focus on whether the current treatment methods for PTTD improve the changes in PTTD postural control during gait.

## Figures and Tables

**Table 1:** Participant demographics.

	PTTD (*n* = 11)	Controls (*n* = 11)	*p* Value
Age (year)	59.1 ± 7.1	55.8 ± 8.9	0.26
Height (m)	1.66 ± 0.12	1.68 ± 0.10	0.64
Weight (kg)	84.2 ± 16.0	76.1 ± 17.1	0.27
Body mass index	30.4 ± 3.5	26.7 ± 4.5	0.07
Gait velocity (m/s)	0.92 ± 0.20	1.13 ± 0.11	0.02
Gender (M/F)	4/7	4/7	-
Affected side (L/R)	9/2	N.A	-

N.A, not available.

**Table 2:** Mean and standard deviations for double stance ratio and COP based parameters.

Variables	PTTD (*n* = 11)	Control (*n* = 11)	*p* Value	Effect Size (D)
Double stance ratio (%)	40.1 (6.1)	32.7 (5.8)	0.009 *	1.2
AP COP excursion (cm)	11.2 (2.9)	13.9 (2.0)	0.020 *	1.1
ML COP excursion (cm)	1.8 (0.7)	1.8 (0.8)	0.965	0.0
AP COP velocity (cm/s)	23.8 (5.7)	33.9 (3.9)	<0.001 *	2.1
ML COP velocity (cm/s)	4.4 (1.3)	7.3 (3.4)	0.018 *	1.2
AP TTC percentage (%)	83.0 (8.2)	71.6 (10.0)	0.009 *	1.2
ML TTC percentage (%)	86.4 (9.3)	80.3 (6.5)	0.094	0.8
ML COP trace (%)	40.6 (11.7)	52.4 (10.6)	0.022 *	1.1

* indicates *p* < 0.05.

## Data Availability

The data presented in this study are available on request from the corresponding author.
